# Lyme disease associated neurological and musculoskeletal symptoms: A systematic review and meta-analysis

**DOI:** 10.1016/j.bbih.2024.100931

**Published:** 2025-01-02

**Authors:** Ganesh Bushi, Ashok Kumar Balaraman, Shilpa Gaidhane, Suhas Ballal, Sanjay Kumar, Mahakshit Bhat, Shilpa Sharma, M Ravi Kumar, Aashna Sinha, Mahalaqua Nazli Khatib, Nishant Rai, Sanjit Sah, Ambanna Yappalparvi, Shailesh Kumar Samal, Doddolla Lingamaiah, Muhammed Shabil

**Affiliations:** a Center for Global Health Research, Saveetha Medical College and Hospital, Saveetha Institute of Medical and Technical Sciences, Saveetha University, Chennai, India; b Research and Enterprise, University of Cyberjaya, Persiaran Bestari, Cyber 11, 63000, Cyberjaya, Selangor, Malaysia; c One Health Centre, Jawaharlal Nehru Medical College, Datta Meghe Institute of Higher Education, Wardha, India; d Department of Chemistry and Biochemistry, School of Sciences, JAIN (Deemed to be University), Bangalore, Karnataka, India; e Department of Allied Healthcare and Sciences, Vivekananda Global University, Jaipur, Rajasthan, 303012, India; f Department of Medicine, National Institute of Medical Sciences, NIMS University Rajasthan, Jaipur, India; g Chandigarh Pharmacy College, Chandigarh Group of Colleges-Jhanjeri, Mohali, 140307, Punjab, India; h Department of Chemistry, Raghu Engineering College, Visakhapatnam, Andhra Pradesh, 531162, India; i Uttaranchal Institute of Pharmaceutical Sciences, Division of Research and Innovation, Uttaranchal University, India; j Division of Evidence Synthesis, Global Consortium of Public Health and Research, Datta Meghe Institute of Higher Education, Wardha, India; k Department of Biotechnology, Graphic Era (Deemed to be University) , Clement Town, Dehradun, 248002, India; l SR Sanjeevani Hospital, Kalyanpur, Siraha, 56517, Nepal; m Department of Paediatrics, Dr. D. Y. Patil Medical College, Hospital and Research Centre, Dr. D. Y. Patil Vidyapeeth, Pune, 411018, Maharashtra, India; n Department of Public Health Dentistry, Dr. D.Y. Patil Dental College and Hospital, Dr. D.Y. Patil Vidyapeeth, Pune, 411018, Maharashtra, India; o School of Pharmaceutical Sciences, Lovely Professional University, Phagwara, India; p Unit of Immunology and Chronic Disease, Institute of Environmental Medicine, Karolinska Institutet, 17177, Stockholm, Sweden; q Noida Institute of Engineering and Technology (Pharmacy Institute), Greater Noida, India; r University Center for Research and Development, Chandigarh University, Mohali, Punjab, India; s Medical Laboratories Techniques Department, AL-Mustaqbal University, 51001, Hillah, Babil, Iraq

**Keywords:** Lyme disease, Tick-borne diseases, Musculoskeletal symptoms, Neurological disabilities, Systematic review, Meta-analysis

## Abstract

**Background and objective:**

Lyme disease, caused by *Borrelia burgdorferi*, presents major health challenges worldwide, leading to serious neurological and musculoskeletal issues that impact patients' lives and healthcare systems. This systematic review and meta-analysis aim to determine the prevalence and link between Lyme disease and these complications, aiming to enhance clinical and public health approaches.

**Methods:**

We systematically searched PubMed, EMBASE, and Web of Science up until April 01, 2024, to find studies reporting the prevalence and severity of neurological and musculoskeletal complications associated with Lyme disease. Screening and data extraction were conducted using Nested Knowledge software. Two independent reviewers performed the quality assessment using the Newcastle-Ottawa Scale. Meta-analyses were performed using R software v4.3, employing a random-effects model.

**Results:**

Out of 3576 records, 17 studies were included, involving 3932 participants. These studies revealed significant prevalence of musculoskeletal symptoms (21.1%) and neurological disabilities (18%) among Lyme disease patients. The analysis showed a notable increase in risk for both complications in individuals with Lyme disease, with pooled Risk Ratios (RR) of 1.82 for musculoskeletal symptoms and 1.64 for neurological disabilities, indicating a significantly higher risk compared to control groups. Although heterogeneity across the studies was high, sensitivity analysis confirmed the consistency of our findings. Additionally, there was evidence of publication bias.

**Conclusion:**

The study reveals significant neurological and musculoskeletal complications in Lyme disease patients, emphasizing the importance of early diagnosis, comprehensive treatment, and supportive care. The noted heterogeneity and potential publication bias highlight the need for transparent research and further study on long-term outcomes.

## 1 Introduction

Lyme disease, a tick-borne illness caused by the spirochete Borrelia burgdorferi and transmitted through infected Ixodes ticks, presents a considerable public health challenge worldwide. Characterized initially by symptoms such as the erythema migrans rash, fever, headache, and fatigue, Lyme disease can progress to severe and chronic stages if left untreated (Shapiro, 2014; Murray et al., 2010). Neurological and musculoskeletal complications associated with Lyme disease vary widely, from focal weakness due to cranial nerve VII palsy to memory impairment, meningitis, encephalitis, cranial neuritis, radiculitis, peripheral neuropathy, and neurocognitive difficulties. Musculoskeletal effects commonly include arthritis affecting the knee, accompanied by joint pain, swelling, arthralgia, myalgia, and muscle discomfort, potentially leading to long-term impairment and reduced quality of life when not promptly diagnosed and treated (Talbot et al., 2023; Smith et al., 2011). These complications, including Lyme neuroborreliosis, arthritis, and persistent post-treatment Lyme disease syndrome, significantly impact morbidity, degrade individuals' quality of life, and place a strain on healthcare systems (Smith et al., 2002; Vázquez et al., 2003; Roos, 2021).

Since it emerged in the United States in the 1970s, Lyme disease has become the most common vector-borne illness in North America and Europe, with over 300,000 new cases reported annually (Talbot et al., 2023; Logigian et al., 1990). While early treatment with antibiotics generally leads to effective resolution and reduces the prevalence of arthritic complications, some patients may experience persistent symptoms even after early-stage treatment, raising questions about the factors contributing to long-term complications (McCarthy et al., 2013). Studies examining these effects show varying results concerning the causes and persistence of musculoskeletal symptoms and neurological disabilities in Lyme disease (Smith et al., 2002; Anan'eva et al., 1995; Seltzer et al., 2000; Zychowski et al., 2024a; Wang et al., 1998).

Although the neurological and musculoskeletal impacts of Lyme disease are widely acknowledged, uncertainties remain regarding the precise prevalence and long-term outcomes of these conditions. Differences in clinical manifestations, diagnostic challenges, and varying treatment responses contribute to inconsistent findings across studies (McCarthy et al., 2013; Wang et al., 1998; Christova and Komitova, 2004; Garrabe et al., 2021; Logina et al., 2006). This variability complicates a comprehensive understanding of Lyme disease's impact the development of effective management strategies. This systematic review and meta-analysis aim to consolidate existing research to clarify the prevalence and associations of neurological and musculoskeletal complications with Lyme disease. Understanding these complexities is essential for healthcare providers in delivering accurate diagnoses, effective treatment, and strategic management.

## 2 Methods

This systematic review and meta-analysis followed the guidelines outlined by the Preferred Reporting Items for Systematic Reviews and Meta-Analyses (PRISMA) (Table S1) (Page et al., 2021). The review protocol was registered with the International Prospective Register of Systematic Reviews (PROSPERO).

### 2.1 Eligibility criteria

Studies involving individuals of any age diagnosed with Lyme disease, as evidenced by clinical diagnosis or laboratory confirmation, were included. We excluded studies focusing on individuals with immunodeficiency disorders or pre-existing musculoskeletal diseases to minimize confounding effects. The primary outcomes were the prevalence and severity of neurological and musculoskeletal complications in Lyme disease patients. Secondary outcomes included the identification of risk factors associated with the development of these complications. We included observational studies (cohort, case-control, and cross-sectional studies) that examined the association between Lyme disease and its neurological and musculoskeletal complications. Case reports, case series, trials, reviews, and studies without clear outcomes related to the complications of interest were excluded (Table S2).

### 2.2 Search strategy

A comprehensive search was conducted in PubMed (MEDLINE), EMBASE, and Web of Science, from inception to January 06, 2024, and subsequently updated on April 01, 2024 without language restrictions. The search strategy combined terms related to Lyme disease (“Lyme disease,” “*Borrelia burgdorferi*”) and terms for neurological and musculoskeletal complications. A detailed search strategy is available in Table S3.

### 2.3 Screening and data extraction

Two reviewers independently screened titles and abstracts for eligibility using Nested Knowledge software, a dedicated web application for conducting systematic reviews (Nested Knowledge, St. Paul, MN, USA). Disagreements were resolved through discussion or, if necessary, consultation with a third reviewer. Potentially relevant studies underwent full-text reviews against the inclusion criteria. The selection process was meticulously documented, including reasons for exclusions. For data extraction, we utilized the ‘tagging’ feature in Nested Knowledge, which captures all variables required to collect information from included studies regarding study characteristics, participant demographics, and outcome measures. This data was organized in Microsoft Excel. Two reviewers independently performed the data extraction, with any discrepancies resolved by consensus.

### 2.4 Quality assessment

The quality of the included studies was assessed using the Newcastle-Ottawa Scale for observational studies (Lo et al., 2014). Two independent reviewers conducted the assessments, with any disagreements resolved through discussion. The scale consists of three main domains: Selection, Comparability, and Outcome or Exposure. The Selection domain includes four items, each worth up to one point, evaluating the representativeness and selection of cohorts, as well as the ascertainment of exposure and the absence of the outcome at the start of the study. Comparability is assessed based on design or analysis, with up to two points awarded. The Outcome or Exposure domain evaluates the outcome assessment, the sufficiency of follow-up, and the adequacy of follow-up or exposure for case-control studies, and cohort studies with each item receiving up to one point. A star system quantifies the quality, allowing a maximum of nine stars to denote high-quality studies. Studies scoring 0–3 stars are considered low quality, 4–6 stars moderate quality, and 7–9 stars high quality. These assessments are performed by two independent reviewers, with any disagreements resolved through discussion to ensure a consensus on the final quality score (Table S4).

### 2.5 Data analysis

All statistical analyses were performed using R software, version 4.3 (Wen et al., 2023). A meta-analysis was conducted employing a random-effects model to assess the Risk Ratio (RR) for musculoskeletal symptoms and neurological disabilities in Lyme disease compared to non-Lyme disease (control) groups, with the aim of pooling results from the included studies. Heterogeneity was assessed using the I^2^ statistic (Langan et al., 2019; Gandhi et al., 2023). Subgroup meta-analyses were performed by combining similar types of musculoskeletal and neurological symptoms. Sensitivity analyses were conducted using the leave-one-out method, where each study was omitted in turn, and the analysis was repeated each time to identify any studies that significantly affected heterogeneity and the pooled results. A quantitative assessment of publication bias was performed using Doi plots and Egger's test to determine the significance of publication bias in the studies (IntHout et al., 2016).

## 3 Results

### 3.1 Literature search

**Fig. 1:**
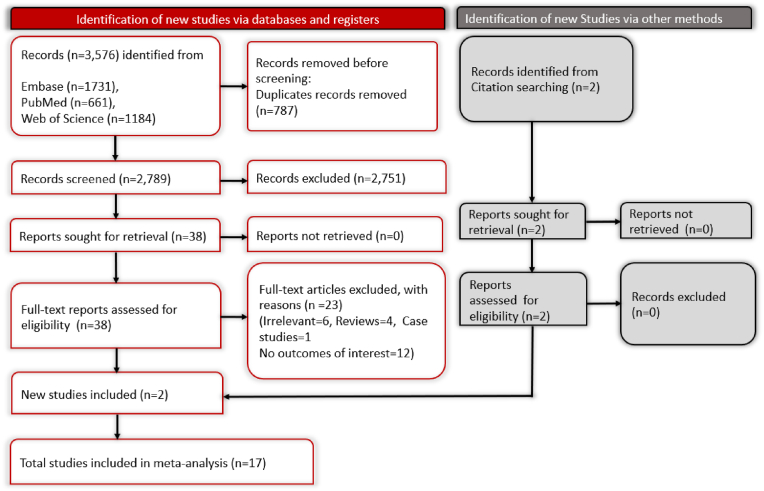
PRISMA flowchart depicting the screening and selection process.

The literature search conducted for this meta-analysis followed structured PRISMA guidelines, as illustrated in Fig. 1. An initial comprehensive search across multiple databases yielded 3576 records. After removing 787 duplicates, we screened 2789 records according to the pre-established exclusion criteria. From this, 2751 non-relevant records were excluded during title and abstract screening. The remaining 38 full-text articles were rigorously evaluated for relevance and suitability according to the study's strict inclusion criteria. Of these, 23 articles were excluded for specific reasons: 6 were reviews, 1 was a commentary on previous literature, 15 focused on non-relevant outcomes, and 1 was a case study. This screening ultimately yielded 15 pertinent studies for in-depth analysis. An additional citation search identified 2 more relevant studies, resulting in a total of 17 studies included in the final meta-analysis.

### 3.2 Summary characteristics of included studies

Table 1 details the characteristics of the included studies that examine the prevalence of musculoskeletal and neurological symptoms in Lyme disease. The research spans several countries, with eight studies from the USA (Smith et al., 2002; Vázquez et al., 2003; Logigian et al., 1990; McCarthy et al., 2013; Seltzer et al., 2000; Wang et al., 1998; Shadick et al., 1994, 1999), Three studies from Poland (Czupryna et al., 2012; Krawczuk et al., 2020; Oczko-Grzesik et al., 2017), two studies from France (Garrabe et al., 2021; Dhôte et al., 2000), one in each from Bulgaria (Christova and Komitova, 2004), UK (Logina et al., 2006), Russia (Anan'eva et al., 1995) and Belgium (Geebelen et al., 2022), reflecting a broad international scope. The study designs vary, including prospective and retrospective cohort studies, community-based longitudinal cohorts, and a cross-sectional study. Specific age details are often undisclosed; however, one study specifically targeted a pediatric population, and another reported a median age of 49 years. The total combined sample size of the participants across all studies is 3932. The studies report a comprehensive range of musculoskeletal symptoms, such as arthralgia, arthritis, synovitis, and muscle pain. Neurological symptoms are also extensively documented, encompassing radiculoneuritis, encephalopathy, facial nerve palsy, memory loss, and various sensory and speech disorders. Outcomes from these studies include the presentation of symptoms and, in more severe cases, hospital admissions. While several studies offer comparative outcomes between Lyme disease patients and control groups, some do not specify a control group. The overall quality of the included studies is considered to range from moderate to high.

**Table 1:** Characteristics of included studies.

Study ID	Country	Study design	Age	Male	Total sample	Musculoskeletal Symptoms	Neurological Symptoms/Disabilities	No. of patients/No. of control
Geebelen et al., 2022 (Geebelen et al., 2022)	Belgium	Prospective cohort study	NA	34.90%	263	Musculoskeletal disease, Joint pain	Fatigue, Memory difficulties, Concentration difficulties, Wording difficulties	120/128
Christova 2004 (Christova and Komitova, 2004)	Bulgaria	Retrospective study	NA	35.70%	1257	Mono- or oligoarthritis, Chronic arthritis	Radiculoneuritis, Cranial neuritis, Encephalopathy, Meningoradiculoneuritis, Myelitis, Ocular manifestation	1257/NA
Dh te 2000 (Dhôte et al., 2000)	France	Retrospective study	44.2	32.30%	170	Arthritis	NA	170/NA
Garrabe et al., 2021 (Garrabe et al., 2021)	France	Retrospective study	8 (median)	NA	26	Cervicalgia, Neck stiffness	Headaches, Asthenia (weakness/fatigue), Facial palsy, Photophobia	26/NA
Czupryna et al., 2012 (Czupryna et al., 2012)	Poland	Prospective study	NA	NA	76	Synovitis, Baker's cyst	NA	60/16
Krawczuk et al., 2020 (Krawczuk et al., 2020)	Poland	Retrospective analysis	NA	55.80%	181	Muscle pain, Neck stiffness, L/S region pain	Vertigo, Short-term memory loss, Sensation disorder: cranial nerves, Sensation disorder: spinal/root, Speech disorders, Vision problems, Meningitis, Facial nerve palsy, Bannwarth's syndrome, Headache	181/NA
Oczko-Grzesik et al., 2017 (Oczko-Grzesik et al., 2017)	Poland	Prospective study	46	49.50%	121	NA	Cognitive disorders, Depression disorder	121/NA
Ananjeva 1995 (Anan'eva et al., 1995)	Russia	Prospective study	NA	NA	86	Arthralgia	Neurologic abnormalities	86/NA
Logina et al., 2006 (Logina et al., 2006)	UK	Retrospective study	50	60%	51	Muscle weakness, Joint involvement, Arthralgias, Swelling, Headache or Meningism	Tremor, Nystagmus, Flaccid paresis	51/NA
Logigian et al., 1990 (Logigian et al., 1990)	USA	Prospective study	49 (median)	51.80%	27	Spinal or radicular pain, Distal paresthesia, Ankle hyporeflexia, Hyperreflexia, Increased muscle tone, Fibromyalgia	Encephalitis, Encephalopathy, Memory loss, Depression, Sleep disturbance, Irritability, Difficulty finding words, Polyneuropathy, Sensory loss, Lower-motor-neuron weakness, Upper-motor-neuron weakness, Fatigue, Headache, Hearing loss, Tinnitus	27/NA
McCarthy 2013 (McCarthy et al., 2013)	USA	Retrospective study	NA	41.20%	218	Joint pain/swelling, Back pain	Cognitive difficulty, Headache, Sleep disturbance, Dizziness/vertigo, Vision problems	218/NA
Setzer 2000 (Seltzer et al., 2000)	USA	Community based longitudinal cohort study	NA	48.40%	424	Swollen joints, Joint or muscle pain, Neck pain	Numbness, Headaches, Memory problems, Fatigue	212/212
Shadick et al., 1994 (Shadick et al., 1994)	USA	Retrospective cohort study	49	NA	81	Arthralgia, Myalgias	Numbness, Coordination difficulties, Seizures, Unusual fatigue, Persistent depression, Concentration difficulties, Emotional lability, Difficulty sleeping	38/43
Shadick et al., 1999 (Shadick et al., 1999)	USA	Retrospective cohort study	>17 years	NA	353	Joint pain, Joint swelling, Muscle aches, Weakness, Neck stiffness	Fatigue, Palpitations, Poor coordination, Headaches, Memory impairment, Poor concentration, Difficulty with word finding, Difficulty sleeping, Numbness	186/167
Smith et al., 2002 (Smith et al., 2002)	USA	Observational cohort study.	15–70 years	NA	118	Myalgia or arthralgia, Stiff neck, Back pain	Headache, Fatigue, Cranial neuritis (facial palsy or trigeminal paresthesia), Other paresthesia	118/NA
Vazquez 2003 (Vázquez et al., 2003)	USA	Cross-sectional study	2–18 years	70%	129	Neck pain, Pains in joints or muscles, Swollen joints	Behavioral changes, Numbness or funny sensations in nerves, Memory problems, Fatigue, Headaches, Activities: School work and attendance, Appetite, Sleeping, Naming objects, Word recall, Academic performance, Organization of ideas	43/86
Wang et al., 1998 (Wang et al., 1998)	USA	Retrospective study	NA	NA	51	Arthralgias, Swollen joints/joint swelling	Memory problems/memory impairment/memory difficulty, Poor concentration/difficulties/attention, Difficulties in word finding/word recall	25/26

### 3.3 Meta-analysis

#### 3.3.1 Prevalence of musculoskeletal symptoms among those diagnosed with Lyme disease

**Fig. 2:**
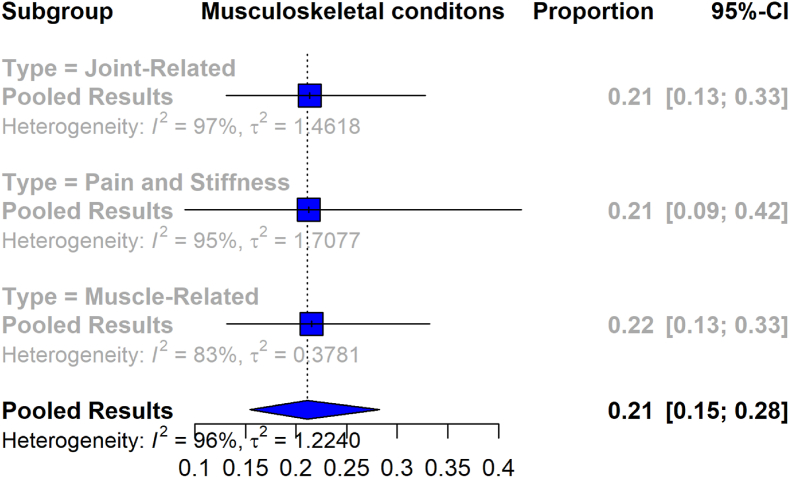
Forest plot depicting the pooled prevalence of Musculoskeletal symptoms in patients diagnosed with Lyme disease.

The prevalence of musculoskeletal symptoms among individuals diagnosed with Lyme disease is depicted in the forest plot in Fig. 2, which categorizes the symptoms into three distinct subgroups across 16 studies. Among the 6651 individuals in the Lyme disease population, the overall pooled prevalence is 21.1% (95% CI [15%, 28%]). The meta-analysis reveals high overall heterogeneity (I^2^ = 96%).

In the subgroup analysis, for joint-related symptoms and conditions, the meta-analysis (13 studies with 4393 patients) indicates a pooled prevalence of 21.3% (95% CI [13%, 33%]), accompanied by a substantial level of heterogeneity (I^2^ = 97%). The subgroup focused on pain and stiffness in specific regions (7 studies with 1309 patients) demonstrates a pooled prevalence of 21% (95% CI [9%, 42%]), with a heterogeneity value of I^2^ = 95%. Muscle-related symptoms and conditions (7 studies with 949 patients) show a pooled prevalence of 22% (95% CI [13%, 33%]), with I^2^ = 83%.

#### 3.3.2 Association between musculoskeletal symptoms and Lyme disease

**Fig. 3:**
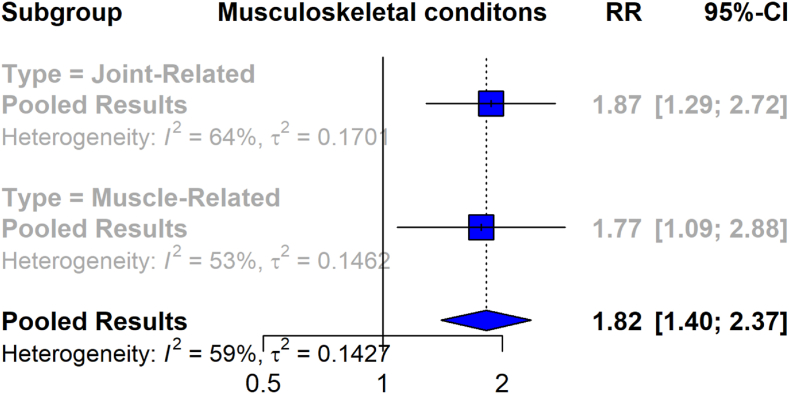
Forest plot depicting the association between Musculoskeletal symptoms Lyme disease.

The forest plot in Fig. 3 illustrates a meta-analysis of the association between musculoskeletal symptoms and Lyme disease, comparing patients with Lyme disease to a control group. Across all 7 studies, encompassing 598 musculoskeletal events among 2211 Lyme disease patients and 314 events among 2139 control participants, the meta-analysis yields an overall RR of 1.82 (95% CI [1.40, 2.37]) with a heterogeneity value of I^2^ = 59%.

The joint-related symptoms and conditions subgroup, which includes data from 7 studies with 1225 Lyme disease patients and 1177 controls, shows a pooled RR of 1.87 (95% CI [1.29, 2.72]), with a heterogeneity value of I^2^ = 64%. For muscle-related symptoms and conditions, the pooled RR from 5 studies, including 986 Lyme disease patients and 962 controls, is 1.77 (95% CI [1.09, 2.88]), with a heterogeneity value of I^2^ = 53%.

#### 3.3.3 Prevalence of neurological conditions among those diagnosed with Lyme disease

**Fig. 4:**
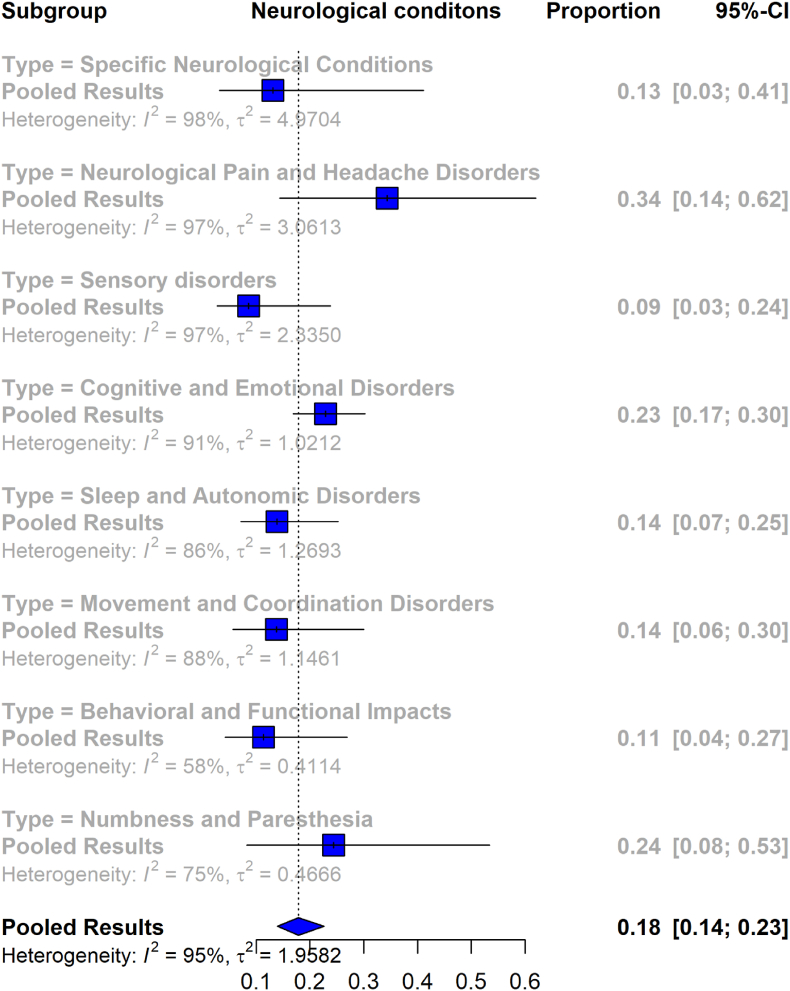
Forest plot illustrating the pooled prevalence of various neurological disabilities in patients diagnosed with Lyme disease.

Fig. 4 illustrates the pooled prevalence of neurological conditions in patients diagnosed with Lyme disease. The overall pooled analysis from 17 studies included a total of 16,357 Lyme disease patients, resulting in a pooled prevalence of 18% (95% CI [14%, 23%]), with high heterogeneity (I^2^ = 95%).

In the subgroup analysis, specific neurological conditions were examined across 7 studies involving 5544 Lyme disease patients, showing a pooled prevalence rate of 13% (95% CI [3%, 41%]), with I^2^ = 97%. For neurological pain and headache disorders, the pooled prevalence from 10 studies with 2526 patients was reported as 34% (95% CI [14%, 62%]), with high heterogeneity (I^2^ = 97%). Sensory disorders, analyzed from 11 studies with 2420 patients, had a pooled prevalence of 9% (95% CI [3%, 24%]), with I^2^ = 97%. The cognitive and emotional disorders subgroup, based on 12 studies involving 3364 Lyme disease patients, demonstrated a pooled prevalence of 23% (95% CI [17%, 30%]), with heterogeneity of I^2^ = 91%. Sleep and autonomic disorders exhibited a pooled prevalence of 14% (95% CI [7%, 25%]) across 5 studies with 1286 patients, showing I^2^ = 88%. Movement and coordination disorders, analyzed from 5 studies with 742 patients, indicated a pooled prevalence of 14% (95% CI [6%, 30%]). Behavioral and functional impacts showed a pooled prevalence of 11% (95% CI [4%, 27%]) in 2 studies involving 199 Lyme disease patients, with moderate heterogeneity (I^2^ = 58%). For numbness and paresthesia, 4 studies with 294 patients revealed a pooled prevalence of 24% (95% CI [8%, 53%]), with heterogeneity of I^2^ = 75%.

#### 3.3.4 Association between neurological disabilities and Lyme disease

**Fig. 5:**
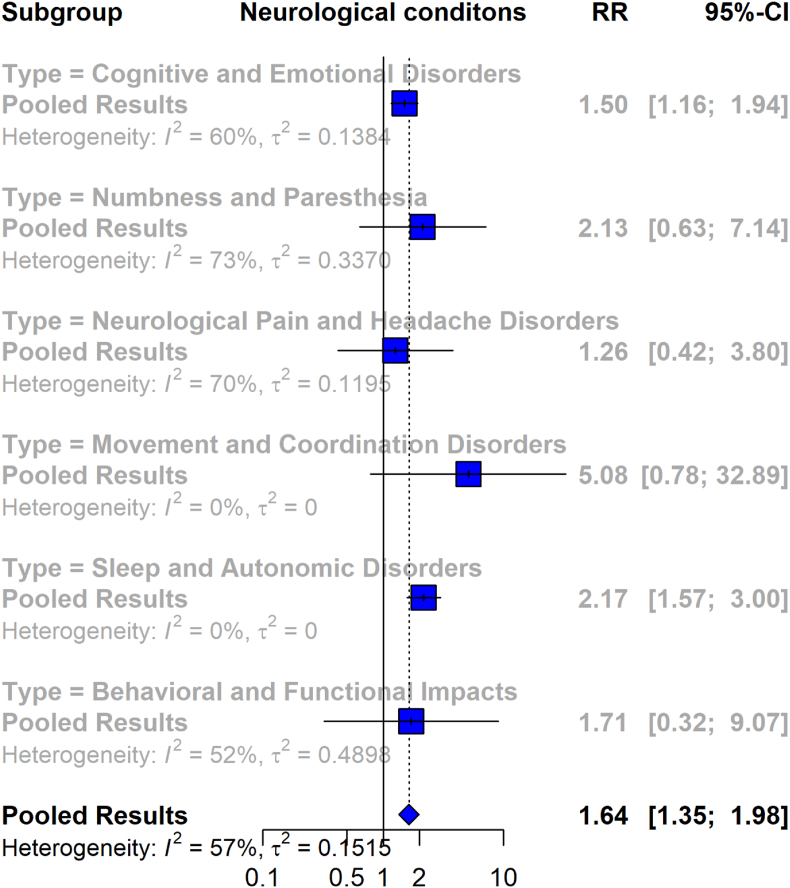
Forest plot representing the association between Lyme disease and neurological disabilities, comparing patients with Lyme disease to control groups across multiple subgroups.

The forest plot in Fig. 5 presents a meta-analysis of the risk of neurological disabilities in patients with Lyme disease compared to a control group. Overall, the meta-analysis includes six studies with 3932 Lyme disease patients and 4311 control participants. The analysis indicates a 64% increased risk of neurological disabilities in Lyme disease patients, with a pooled RR of 1.64 (95% CI [1.35, 1.98]), and moderate heterogeneity (I^2^ = 57%).

In the subgroup analysis, for cognitive and emotional disorders, the pooled RR is 1.50 (95% CI [1.16, 1.94]) across six studies, indicating a significantly higher risk for Lyme disease patients compared to controls, with heterogeneity of I^2^ = 60%. In the numbness and paresthesia subgroup, the pooled RR is 2.13 (95% CI [0.63, 7.14]) across four studies, with I^2^ = 73%. Neurological pain and headache disorders are represented in three studies, showing a pooled RR of 1.26 (95% CI [0.42, 3.80]), with high heterogeneity (I^2^ = 70%). Movement and coordination disorders, assessed in two studies, yield an RR of 5.08 (95% CI [0.78, 32.89]), with I^2^ = 0%. Sleep and autonomic disorders, based on three studies, show a pooled RR of 2.17 (95% CI [1.57, 3.00]), with no observed heterogeneity (I^2^ = 0%). Behavioral and functional impacts, assessed in a single study, have an RR of 1.71 (95% CI [0.32, 9.07]), with moderate heterogeneity (I^2^ = 52%).

### 3.4 Sensitivity analysis

A sensitivity analysis was conducted using the leave-one-out approach to examine the influence of individual studies on the pooled estimate of musculoskeletal symptoms and neurological disabilities in Lyme disease patients. The recalculated pooled proportions remained consistent even when each study was sequentially omitted, suggesting that no single study exerted disproportionate influence on the overall result. Despite a high heterogeneity I^2^ value of 96% was noted across all recalculated estimates, indicating that the variability in the results was not solely attributable to any single study but rather to other factors such as differences in study design or patient populations. In essence, the sensitivity analysis underscores the robustness of the findings (Figs. S1 and S2).

### 3.5 Publication bias

In assessing publication bias for the prevalence of musculoskeletal symptoms and neurological disabilities in Lyme disease, the analysis revealed varying potential biases. The Doi plot for musculoskeletal symptoms indicated a possible publication bias with an LFK index of −2.15. In the case of neurological disabilities, the LFK index was −2.34, which strongly suggests publication bias. This is further substantiated by a significant Egger's test result, with a p-value of 0.0053, indicating the presence of publication bias. The evidence suggests that the true effect may be skewed due to the selective publication of studies with larger effects, necessitating a critical consideration of the findings (Figs. S3 and S4).

## 4 Discussion

This systematic review and meta-analysis highlight the substantial burden of neurological and musculoskeletal complications associated with Lyme disease. Across the 17 studies involving 3632 individuals, we found pooled prevalence rates of 21% for musculoskeletal symptoms and 18% for neurological disabilities among patients diagnosed with Lyme disease. These associations are further supported by pooled RRs, indicating that individuals with Lyme disease are more likely to experience musculoskeletal symptoms (RR = 1.82) and have a 64% increased risk of neurological disabilities (RR = 1.64) compared to those without the disease. This evidence demonstrates the broad-reaching effects of Lyme disease on patients, extending significantly beyond the initial infection phase and underscoring the need for a multidisciplinary approach to its management.

Interestingly, movement and coordination disorders, though represented in only two studies (Shadick et al., 1994, 1999), showed a notably high Risk Ratio (RR = 5.08, 95% CI [0.78, 32.89]), suggesting a potential increase in the risk of these complications among Lyme disease patients. Despite the heterogeneity being reported as I^2^ = 0% for these studies, the wide confidence interval suggests that further focused research is necessary to better understand these complications. The connection between Lyme disease and these musculoskeletal and neurological complications likely involves complex pathophysiological mechanisms. Infection with *Borrelia burgdorferi* can trigger an inflammatory immune response, potentially producing autoantibodies that mistakenly target the body's own tissues, leading to pain and cognitive dysfunction (Halperin, 2014; Tjernberg et al., 2017). Additionally, the bacteria can directly invade tissues, resulting in arthritis and various neurological impairments (Skar et al., 2024). Other tick-borne illnesses, such as alpha-gal syndrome, present different symptoms, including allergic musculoskeletal discomfort, which further emphasizes the diverse clinical manifestations of tick-borne diseases (Zychowski et al., 2024b).

Our findings align with those of Cairns et al. (2005) (Cairns and Godwin, 2005), who documented post-Lyme disease complications, including neurocognitive impairments like memory issues and concentration difficulties, which are distinct from symptoms associated with conditions like fibromyalgia. This review identified fatigue, joint or muscle pain, and swollen joints as notably common symptoms among Lyme disease patients. However, some neurocognitive symptoms, such as judgment difficulties and object naming, were less frequently reported, possibly due to the limited number of studies examining these specific outcomes. Furthermore, Garrabe et al. (2021) (Garrabe et al., 2021) documented significant cerebrospinal fluid abnormalities in children with Lyme neuroborreliosis, especially those aged 5–8 years, highlighting the neurological severity of Lyme disease in pediatric populations. This progression from early symptoms, such as erythema migrans, to complex neurological conditions like encephalopathy and polyneuropathy, underscores the serious, chronic implications of Lyme disease. Conversely, Zychowski et al. (2024) (Zychowski et al., 2024a) found no significant association between other tick-borne diseases and chronic musculoskeletal symptoms in a North Carolina cohort, although they observed a correlation between elevated α-gal IgE levels and knee pain, emphasizing the variability in the impact of tick-borne illnesses on musculoskeletal health.

Despite these insights, this review has limitations. The high heterogeneity observed across studies affects the generalizability of our results. Additionally, the potential for publication bias from Doi plots and Egger's test indicates a possible underrepresentation of smaller or less impactful studies. This bias may mean that the actual impact of Lyme disease on neurological and musculoskeletal health could be more significant than reported, underscoring the importance of comprehensive and transparent reporting in future studies.

The strengths of this review include a thorough analysis of the wide range of musculoskeletal and neurological complications associated with Lyme disease. Musculoskeletal outcomes ranged from generalized joint pain to specific conditions like chronic arthritis, while neurological complications included cognitive and emotional disorders, sensory impairments, and movement disturbances. The sensitivity analysis conducted supports the robustness of these findings, suggesting that the observed associations are stable and not unduly influenced by any single study.

In terms of prevention and treatment, early diagnosis and immediate intervention remain crucial in reducing the risk of musculoskeletal and neurological complications associated with Lyme disease. Primary prevention measures, such as avoiding tick-infested areas, using repellents, wearing protective clothing, and promptly removing ticks, are essential (Krawczuk et al., 2020). Diagnostic methods, including ELISA and Western blot tests, can occasionally produce inaccurate results, but lumbar punctures and imaging can aid in confirming severe neurological issues (Geebelen et al., 2022). Treatment primarily involves antibiotics, with additional options like pain relievers and rehabilitation as necessary to improve function (Czupryna et al., 2012). Regular follow-ups for those with persistent symptoms post-treatment are also recommended. Current research is focused on developing new treatments and vaccines, highlighting the ongoing need for awareness and proactive measures to manage this disease effectively.

Future research should prioritize the standardization of diagnostic criteria for Lyme disease complications and strive for transparency to minimize publication bias. Longitudinal studies are essential to investigate the long-term outcomes of these complications and develop targeted management strategies. Understanding the pathophysiological mechanisms underlying these complications will further enhance treatment efficacy and improve patient outcomes, particularly concerning risk factors and long-term impacts. Additionally, public health initiatives should aim to increase awareness of Lyme disease's chronic nature, promote preventive measures, and provide support for affected individuals, focusing on early diagnosis, comprehensive treatment plans, and strong supportive care strategies.

## 5 Conclusion

This review revealed a significant prevalence and elevated risk of neurological and musculoskeletal complications in individuals diagnosed with Lyme disease. These findings underscore the importance of early diagnosis, effective treatment, and supportive care to manage these complications. The observed heterogeneity and indications of publication bias highlight the need for more rigorous and transparent research practices. Future studies should prioritize standardizing diagnostic criteria, investigating long-term outcomes, and clarifying the underlying pathophysiological mechanisms. Improving our understanding and management of Lyme disease will lead to better patient outcomes and help reduce the healthcare burden associated with this tick-borne illness.

## CRediT authorship contribution statement

**Ganesh Bushi:** Project administration, Data curation, Conceptualization. **Ashok Kumar Balaraman:** Conceptualization, Data curation, Formal analysis, Methodology, Writing – review & editing. **Shilpa Gaidhane:** Formal analysis, Data curation, Conceptualization. **Suhas Ballal:** Methodology, Investigation, Conceptualization. **Sanjay Kumar:** Methodology, Investigation, Conceptualization. **Mahakshit Bhat:** Writing – review & editing, Writing – original draft, Validation, Supervision. **Shilpa Sharma:** Writing – review & editing, Writing – original draft, Software. **M Ravi Kumar:** Software, Resources, Project administration. **Aashna Sinha:** Project administration. **Mahalaqua Nazli Khatib:** Writing – review & editing, Writing – original draft, Conceptualization. **Nishant Rai:** Writing – review & editing, Writing – original draft, Supervision, Data curation, Conceptualization. **Sanjit Sah:** Visualization, Validation, Software, Conceptualization. **Ambanna Yappalparvi:** Writing – original draft, Validation, Supervision. **Shailesh Kumar Samal:** Conceptualization, Data curation, Resources, Supervision, Visualization, Writing – original draft, Writing – review & editing. **Doddolla Lingamaiah:** Conceptualization, Data curation, Investigation. **Muhammed Shabil:** Supervision, Methodology, Investigation.

## Ethical approval

Not required.

## Funding

This study received no funding

## Declaration of competing interest

The authors report no conflict of interest.

## Data Availability

Data will be made available on request.

## References

[bib1] Anan'eva L.P., Skripnikova I.A., Barskova V.G., Steere A.C. (1995). [The clinical and serological manifestations of Lyme disease in Russia]. Ter. Arkh..

[bib2] Cairns V., Godwin J. (2005). Post-Lyme borreliosis syndrome: a meta-analysis of reported symptoms. Int. J. Epidemiol..

[bib3] Christova I., Komitova R. (2004). Clinical and epidemiological features of Lyme borreliosis in Bulgaria. Wien Klin. Wochenschr..

[bib4] Czupryna P., Moniuszko A., Czeczuga A., Pancewicz S., Zajkowska J. (2012). Ultrasonographic evaluation of knee joints in patients with Lyme disease. Int. J. Infect. Dis..

[bib5] Dhôte R., Basse-Guerineau A., Beaumesnil V., Christoforov B., Assous M. (2000). Full spectrum of clinical, serological, and epidemiological features of complicated forms of Lyme borreliosis in the Paris, France, area. Eur. J. Clin. Microbiol. Infect. Dis..

[bib6] Gandhi A.P., Shamim M.A., Padhi B.K. (2023). Steps in undertaking meta-analysis and addressing heterogeneity in meta-analysis. The Evidence.

[bib7] Garrabe E., Dubois D., Chaix Y., Baudou E., Cheuret E., Brehin C. (2021). Lyme neuroborreliosis in pediatrics: a retrospective, descriptive study in southwest France. Arch. Pediatr..

[bib8] Geebelen L., Lernout T., Devleesschauwer B., Kabamba-Mukadi B., Saegeman V., Belkhir L. (2022). Non-specific symptoms and post-treatment Lyme disease syndrome in patients with Lyme borreliosis: a prospective cohort study in Belgium (2016-2020). BMC Infect. Dis..

[bib9] Halperin J.J. (2014). Lyme disease: neurology, neurobiology, and behavior. Clin. Infect. Dis..

[bib10] IntHout J., Ioannidis J.P., Rovers M.M., Goeman J.J. (2016). Plea for routinely presenting prediction intervals in meta-analysis. BMJ Open.

[bib11] Krawczuk K., Czupryna P., Pancewicz S., Ołdak E., Król M., Moniuszko-Malinowska A. (2020). Comparison of neuroborreliosis between children and adults. Pediatr. Infect. Dis. J..

[bib12] Langan D., Higgins J.P., Jackson D., Bowden J., Veroniki A.A., Kontopantelis E. (2019). A comparison of heterogeneity variance estimators in simulated random‐effects meta‐analyses. Res. Synth. Methods.

[bib13] Lo C.K.-L., Mertz D., Loeb M. (2014). Newcastle-Ottawa Scale: comparing reviewers' to authors' assessments. BMC Med. Res. Methodol..

[bib14] Logigian E.L., Kaplan R.F., Steere A.C. (1990). Chronic neurologic manifestations of Lyme disease. N. Engl. J. Med..

[bib15] Logina I., Krumina A., Karelis G., Elsone L., Viksna L., Rozentale B., Donaghy M. (2006). Clinical features of double infection with tick-borne encephalitis and Lyme borreliosis transmitted by tick bite. J. Neurol. Neurosurg. Psychiatry.

[bib16] McCarthy M.L., Reece R., Vargas S.E., Johnson J., Adelson-Mitty J., Flanigan T. (2013). Lessons learned from a Rhode Island academic out-patient lyme and tick-borne disease clinic. R. I. Med. J..

[bib17] Murray T.S., Shapiro E.D., disease Lyme (2010). Clin. Lab. Med..

[bib18] Oczko-Grzesik B., Kepa L., Puszcz-Matlinska M., Pudlo R., Zurek A., Badura-Glabik T. (2017). Estimation of cognitive and affective disorders occurrence in patients with Lyme borreliosis. Ann. Agric. Environ. Med..

[bib19] Page M.J., McKenzie J.E., Bossuyt P.M., Boutron I., Hoffmann T.C., Mulrow C.D. (2021). The PRISMA 2020 statement: an updated guideline for reporting systematic reviews. Int. J. Surg..

[bib20] Roos K.L. (2021). Neurologic complications of lyme disease. Continuum.

[bib21] Seltzer E.G., Gerber M.A., Cartter M.L., Freudigman K., Shapiro E.D. (2000). Long-term outcomes of persons with Lyme disease. JAMA.

[bib22] Shadick N.A., Phillips C.B., Logigian E.L., Steere A.C., Kaplan R.F., Berardi V.P. (1994). The long-term clinical outcomes of Lyme disease. A population-based retrospective cohort study. Ann. Intern. Med..

[bib23] Shadick N.A., Phillips C.B., Sangha O., Logigian E.L., Kaplan R.F., Wright E.A. (1999). Musculoskeletal and neurologic outcomes in patients with previously treated Lyme disease. Ann. Intern. Med..

[bib24] Shapiro E.D. (2014). Borrelia burgdorferi (Lyme disease). Pediatr. Rev..

[bib25] Skar G.L., Blum M.A., Simonsen K.A. (2024).

[bib26] Smith R.P., Schoen R.T., Rahn D.W., Sikand V.K., Nowakowski J., Parenti D.L. (2002). Clinical characteristics and treatment outcome of early Lyme disease in patients with microbiologically confirmed erythema migrans. Ann. Intern. Med..

[bib27] Smith B.G., Cruz A.I., Milewski M.D., Shapiro E.D. (2011). Lyme disease and the orthopaedic implications of lyme arthritis. J. Am. Acad. Orthop. Surg..

[bib28] Talbot N.C., Spillers N.J., Luther P., Flanagan C., Soileau L.G., Ahmadzadeh S. (2023). Lyme disease and post-treatment lyme disease syndrome: current and developing treatment options. Cureus.

[bib29] Tjernberg I., Hamsten C., Apostolovic D., van Hage M. (2017). IgE reactivity to α-Gal in relation to Lyme borreliosis. PLoS One.

[bib30] Vázquez M., Sparrow S.S., Shapiro E.D. (2003). Long-term neuropsychologic and health outcomes of children with facial nerve palsy attributable to Lyme disease. Pediatrics.

[bib31] Wang T.J., Sangha O., Phillips C.B., Wright E.A., Lew R.A., Fossel A.H. (1998). Outcomes of children treated for Lyme disease. J. Rheumatol..

[bib32] Wen X., Thomas M.A., Liu L., Moe A.A., Duong P.H., Griffiths M.E., Munlyn A.L. (2023). Association between maternal e-cigarette use during pregnancy and low gestational weight gain. Int. J. Gynaecol. Obstet..

[bib33] Zychowski D.L., Alvarez C., Abernathy H., Giandomenico D., Choudhary S.K., Vorobiov J.M. (2024). Tick-borne disease infections and chronic musculoskeletal pain. JAMA Netw. Open.

[bib34] Zychowski D.L., Alvarez C., Abernathy H., Giandomenico D., Choudhary S.K., Vorobiov J.M. (2024). Tick-borne disease infections and chronic musculoskeletal pain. JAMA Netw. Open.

